# Polymer-based antimicrobial strategies for periodontitis

**DOI:** 10.3389/fphar.2024.1533964

**Published:** 2025-01-06

**Authors:** Jiajia Chen, Shujun Dong

**Affiliations:** The First Outpatient Department, Jilin Provincial Key Laboratory of Tooth Development and Bone Remodeling, School and Hospital of Stomatology, Jilin University, Changchun, China

**Keywords:** antimicrobial, polymer, periodontitis, nanotherapies, mechanisms

## Abstract

Periodontitis is a chronic inflammatory condition driven by plaque-associated microorganisms, where uncontrolled bacterial invasion and proliferation impair host immune responses, leading to localized periodontal tissue inflammation and bone destruction. Conventional periodontal therapies face challenges, including incomplete microbial clearance and the rise of antibiotic resistance, limiting their precision and effectiveness in managing periodontitis. Recently, nanotherapies based on polymeric materials have introduced advanced approaches to periodontal antimicrobial therapy through diverse antimicrobial mechanisms. This review explored specific mechanisms, emphasizing the design of polymer-based agents that employ individual or synergistic antimicrobial actions, and evaluated the innovations and limitations of current strategies while forecasting future trends in antimicrobial development for periodontitis.

## 1 Introduction

As a prevalent biofilm-associated oral disease, periodontitis presents a significant public health concern (Sczepanik et al., 2020). This condition, driven by microbial factors and host responses, leads to gingival inflammation, periodontal pocket formation, and alveolar bone loss, ultimately resulting in tooth loss if untreated (Yuan et al., 2023). Early prevention and intervention are essential to slow disease progression and maintain oral health (Janakiram and Dye, 2020; Pattamatta et al., 2024). The primary periodontal treatment methods—mechanical debridement through scaling and root planning—aim to reduce bacterial load, alleviate inflammation, and inhibit bone loss, but its effectiveness is often limited by difficult-to-reach infection sites (Petersilka et al., 2002; Arweiler et al., 2017). Conventional antibiotics, which inhibit bacterial growth by targeting key biological processes (Saikia and Chetia, 2024), face limitations in periodontitis due to the need for high doses to penetrate biofilms, risking microbiome disruption, fungal overgrowth, allergic reactions, and other adverse effects (Nakajima et al., 2021; Yu et al., 2022; Herrera et al., 2023). Additionally, the rise of antimicrobial resistance—through mechanisms such as efflux pump activation and membrane alteration—further complicates treatment efficacy (Abouelhadid et al., 2020; Klenotic et al., 2020; Darby et al., 2022). These challenges underscore the need for innovative periodontal therapies capable of overcoming resistance and enhancing therapeutic outcomes.

Periodontitis arises primarily from the destructive symbiotic relationship between dental plaque biofilms and the immune system of host (Lamont et al., 2018). The bacterial hydrolysis of proteins and the resulting inflammatory response create an acidic microenvironment that selectively promotes the growth of periodontitis-associated bacteria (Xiu et al., 2022; Hsiao et al., 2014). This cycle of bacterial proliferation leads to the characteristic loss of soft and hard tissue in periodontitis, emphasizing the need for targeted antimicrobial therapies to interrupt this pathological progression (Li et al., 2024). This review focused on the latest therapeutic strategies using polymer-based macromolecular materials to combat periodontitis-associated bacteria. We summarized the primary antimicrobial mechanisms of novel agents for periodontitis treatment and discussed innovative polymeric material designs that leverage these mechanisms. Finally, we addressed the challenges in antimicrobial therapy, offering insights for future developments in the field.

## 2 Polymer-based antimicrobial strategies

Conventional antibiotics kill bacteria by disrupting bacterial membranes and walls or by interfering with protein synthesis and metabolic processes (Lewis et al., 2024). However, pure drugs are prone to isomerization and degradation in aqueous environments, which diminish their antimicrobial efficacy. To overcome this, complexes are introduced to enhance stability and preserve potency (Lam et al., 2018; Kaupbayeva and Russell, 2020; Haktaniyan and Bradley, 2022). For example, metronidazole (MTZ) has been encapsulated in solid lipid nanoparticles (SLN) or electrostatically spun into triple-layered eccentric side-by-side fibrous structures for controlled release, improving the efficacy of periodontitis treatment (Zhao et al., 2024). Polymeric drug delivery systems enhance the solubility, reduce toxicity, and increase the stability of antimicrobial agents. These systems also enable controlled and programmed drug release in response to specific microenvironmental changes. Critical factors in polymer design, such as particle size, surface charge, hydrophobicity, mucosal adhesion, and targeted ligands, influence the ability to penetrate biofilms and effectively kill bacteria (Huo et al., 2016; Jiang et al., 2023; Ju et al., 2023). Novel antimicrobial polymers are strategically designed to target different bacterial mechanisms, and the combination of multiple mechanisms allows for precise bacterial localization and efficient destruction, offering a promising solution for periodontal therapy (Shi et al., 2021a; Zou et al., 2015; De et al., 2022). Below, we discussed novel antimicrobial strategies involving polymers, categorized by the antimicrobial mechanisms.

### 2.1 Mechanisms of membrane disruption

Membrane integrity is essential for bacterial survival, and electrostatic interactions are key in disrupting bacterial membranes. Various recognition units—such as antibodies, biomolecules, chemical moieties, and functional nanomaterials—have been developed based on biological and chemical characteristics of bacterial surfaces (Yin et al., 2024). These recognition units fall into two main categories: non-specific recognition and ligand-receptor-specific recognition.

#### 2.1.1 Non-specific recognition based on electrostatic interactions

Distinguished from specific recognition that occurs for specific biomolecules, non-specific recognition usually refers to molecules interacting with each other through non-covalent bonds, such as electrostatic interactions. Electrostatic interactions refer to the attraction or repulsion between charged groups. The main advantage of using non-specific interactions is that spatio-temporal controlled release and precise targeting of drugs can be achieved from an antimicrobial platform, ultimately leading to efficient antimicrobials (Adibnia et al., 2020). Bacterial membrane surfaces are rich in negative charges, primarily due to the extracellular glycosylation of phospholipids and membrane proteins. The interaction between nanomedicines and bacterial membranes is more complex than simple electrostatic attraction. Nanomedicines with different charge characteristics exhibit distinct mechanisms of action and endocytosis pathways. Positively charged nanodrugs selectively interact with negatively charged lipids through electrostatic forces, enhancing adsorption and endocytosis. This process effectively crosses the bacterial membrane, inducing tension that leads to membrane deformation and rupture (Yuan et al., 2020).

Polyamino acids exhibit distinct properties dictated by the structure of their side chains (Shen et al., 2018). Three alternating amino acid copolymers—Orn-Ser, Orn-Gly, and Orn-Val—were synthesized using solid-phase peptide technology. The diverse side chains demonstrated excellent biocompatibility, broad-spectrum antimicrobial activity, and selective antimicrobial properties (Ma et al., 2023). ε-Polylysine, a naturally occurring cationic peptide, exerts potent antimicrobial effects by disrupting bacterial membranes, inducing oxidative stress, and modulating gene expression (Li et al., 2019; Dima et al., 2020; Huang et al., 2024; Rao et al., 2024). To enhance antimicrobial efficacy, a novel double-crowned vesicle system, formed by the co-assembly of two block copolymers (PCL-b-P (Lys-co-Phe) and PEO-b-PCL), was designed for periodontitis treatment. The PEO crown imparted protein-repelling properties, enabling penetration of extracellular polymeric substances in biofilms, while the P(Lys-co-Phe) crown provided positive charge, bacterial membrane penetration, and broad-spectrum antimicrobial effects (Xi et al., 2019).

α-Lipoic acid (LA), a mitochondrial coenzyme, confers antioxidant and anti-inflammatory effects via its disulfide ring structure (Yu et al., 2024). LA reduces oxidative stress and apoptosis by promoting osteogenic marker expression through the NOX4, NF-κB, JNK, and PI3K/AKT pathways, ultimately mitigating periodontal bone loss (Lu et al., 2017b). Additionally, LA induces morphological changes and dysfunction in bacterial membranes to achieve antimicrobial effects (Shi et al., 2016; Mu et al., 2022). Wet-reactive PolyLA-GelMA elastic patches exhibited strong adhesion to gingival tissues through intermolecular interactions, making them suitable for filling periodontal defects and promoting alveolar bone regeneration. *In vitro* studies confirmed the patches’ excellent hemocompatibility, antibacterial properties, and ROS-scavenging ability (Qi et al., 2024). Gelatin, a natural macromolecular polymer that mimics the extracellular matrix (ECM) and contains RGD sequences, is commonly used to create natural gels. Gelatin methacryloyl (GelMA) hydrogels possess intrinsic antimicrobial properties, which are enhanced by quaternary ammonium groups and glycidyl-trimethylammonium chloride (GTMAC), effectively inhibiting bacterial enzymes, such as gingival protease (Vargas-Alfredo et al., 2022). Natural polyphenol microspheres were innovatively fabricated by combining T-NPs and poloxamer to create a pH- and temperature-sensitive antibiotic delivery platform, enabling *in situ* slow release of T-NPs at periodontitis lesions. This approach mitigated oxidative stress and inhibited oral pathogenic bacteria (Qi et al., 2022).

The suitability of Chitosan for periodontal applications stems from its exceptional biocompatibility, biodegradability, selective permeability, and antimicrobial activity. The positively charged, low-molecular-weight chitosan binds to negatively charged teichoic acid or lipopolysaccharide through electrostatic interactions, enhancing membrane permeability to exert extracellular antimicrobial effects (Ke et al., 2021). Chitosan oligomers cross bacterial cell wall, inhibiting DNA/RNA transcription, protein synthesis, and mitochondrial function, thus demonstrating intracellular antimicrobial activity (Raafat and Sahl, 2009; Kravanja et al., 2019). Furthermore, chitosan interferes with bacterial copolymerization (Tan et al., 2018). Chemically modified chitosan derivatives exhibit enhanced antimicrobial activity and water solubility. The introduction of quaternary ammonium groups enhances the cationic properties of chitosan and improves its solubility in aqueous and alkaline solutions (Tan et al., 2013). These modifications include the attachment of functional groups bind to anionic sites on microbial membrane surfaces, which not only promotes agglutination and inhibits microbial proliferation, but also disrupts bacterial membranes and leads to leakage of intracellular DNA and RNA, thereby improving antimicrobial efficacy (Murotomi et al., 2023). Diabetic patients are particularly vulnerable to periodontal disease due to the immune system suppression caused by hyperglycemia, necessitating effective antimicrobial solutions. A photo-crosslinked chitosan hydrogel self-regulates the release of therapeutic drugs based on glucose levels, enabling both glucose detection and pH-responsive drug release within the periodontal environment. This system provided a controlled drug delivery mechanism for chitosan (CS)-methacrylamide (CM) formulations (Liu et al., 2019). It is well-established that chitosan modified with quaternary ammonium groups demonstrates superior antimicrobial activity compared to unmodified chitosan. TMC-Lip-DOX nanoparticle, which conjugate quaternary ammonium-modified chitosan (N,N,N-trimethylchitosan) with liposomes and doxycycline, forms a pH-responsive system effective against dental biofilms. The nanoparticle accumulates in acidic environments via electrostatic interactions, penetrate biofilms, degrade extracellular polymers, and enhance doxycycline’s antimicrobial effect, offering a promising strategy for preventing and treating periodontitis (Hu et al., 2019).

To achieve precise antimicrobial effects, introducing a local stimulus-response mechanism into drug delivery systems is a common targeted strategy. *Streptococcus lactis* peptides, with inherent antimicrobial properties, are encapsulated in sodium caseinate (SC) carriers through strong interactions with anionic alginate molecules. This nanocarrier system not only enhances antimicrobial activity but also promotes biofilm elimination and imparts pH responsiveness (Niaz et al., 2020). Liu et al. developed a novel functional peptide module (FPM) consisting of a short antimicrobial peptide (SAMP) flanked by two anchoring peptides containing arginine protease (Rgp)-specific splice sites. This design uses the gingipain secreted by *Porphyromonas gingivalis* as a responsive stimulus, resulting in both intense gingipain reactivity and potent inhibition of *Porphyromonas gingivalis* growth (Liu et al., 2021).

Antimicrobial peptides (AMPs) offer significant advantages over traditional antibiotics in oral antimicrobial therapies. Known for their lysine- and arginine-rich properties, as well as their amphiphilic structures, AMPs inhibit bacterial dehydrogenase activity and disrupt bacterial membranes through electrostatic interactions and hydrogen bonding. This leads to membrane rupture and bacterial cell death (Gao et al., 2023). AMPs also interfere with peptidoglycan synthesis by binding to precursor molecules involved in bacterial cell wall formation or by directly interacting with peptidoglycan and amino acids. In particular, for Gram-negative bacteria, AMPs bind to negatively charged lipopolysaccharides in the outer membrane, forming peptide-lipid complexes that generate transmembrane channels, compromising cell membrane integrity. Additionally, acidic phospholipids in the bacterial membrane interact electrostatically with cationic AMPs, while the hydrophobic regions of the peptides accumulate on the amphiphilic phospholipid surface, further disrupting the membrane structure (Yount and Yeaman, 2013; Luo and Song, 2021).

**FIGURE 1:**
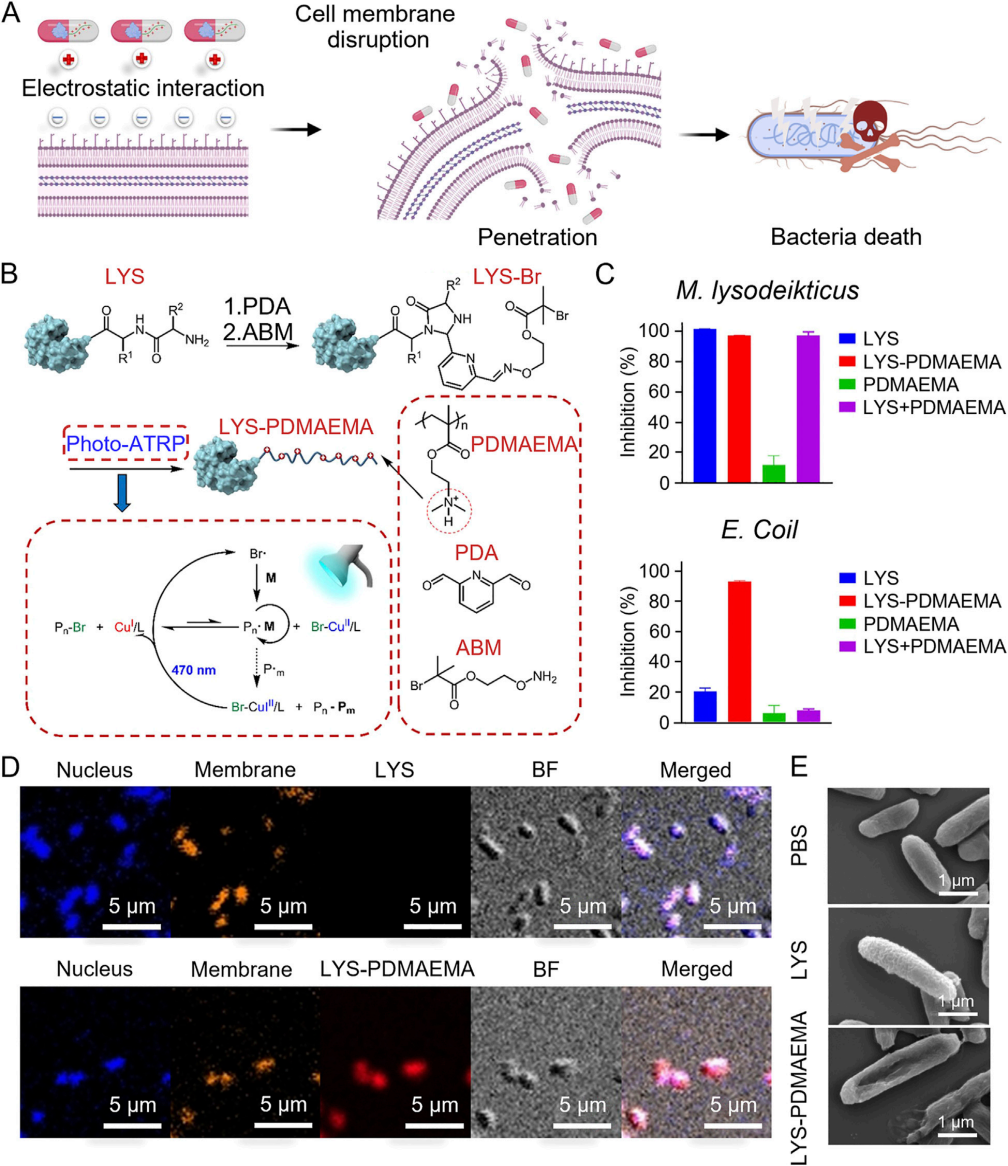
**(A)** Scheme of the antibacterial mechanism of LYS-PDMAEMA. **(B)** Scheme of the synthesis of LYS-PDMAEMA. **(C)** Antimicrobial activity of LYS-PDMAEMA against *M. lysodeikticus* and *E. coli*. **(D)** CLSM images of *E. coli* incubated with Cy5-labeled LYS and LYS-PDMAEMA (red). The nucleus was stained with DAPI (blue). The membrane was stained with Dil (yellow). **(E)** SEM images of *E. coli* treated with LYS and LYS-PDMAEMA (Zhang et al., 2022). Copyright ^©^ 2022 American Chemical Society.

Plant antimicrobial peptides (PMAMPs) with a circular or hairpin structure exhibit enhanced biofilm penetration and antimicrobial activity when combined with matrix-degrading enzymes. These PMAMPs consist of a green fluorescence protein (GFP)-fusion peptide and the protein drug protegrin-1 (PG-1), which rapidly kill bacteria within 1 h of local exposure at low concentrations. The permeabilization mechanism is primarily driven by changes in bacterial surface charge, PG-1 penetration of the lipid bilayer, and interactions with negatively charged teichoic and lipoteichoic acids (Liu et al., 2016). Arginine-rich β-hairpin peptides self-assemble into hydrogels that lyse bacteria through guanidine-mediated interactions with bacterial membranes, independent of antibiotics. The antimicrobial potency of these hydrogels is significantly increased by higher arginine content. To balance antimicrobial activity with reduced cytotoxicity and hemolysis, the PEP6R peptide was synthesized by substituting an arginine residue in the PEP8R peptide. At a concentration of 1.5 wt%, PEP6R self-assembled into a hydrogel of moderate hardness that effectively eradicated bacteria (Veiga et al., 2012). Lysozyme (LYS) effectively kills Gram-positive bacteria by disrupting the β-1,4-glycosidic bonds in the bacterial cell wall and inducing membrane rupture. However, its activity against Gram-negative bacteria is relatively weak. To enhance both the antimicrobial efficacy and stability of LYS, 2,2-pyridinedicarboxaldehyde (PDA) and the initiator ABM were introduced at the N-terminus of LYS, forming a LYS-PDMAEMA conjugate. This conjugate exhibited superior activity against Gram-positive bacteria of *M. lysodeikticus* and Gram-negative bacteria of *E. coli.* The distribution of the red fluorescent Cy5-labeled conjugate on the bacterial surface and within the bacteria highlighted the proposed antibacterial mechanism: the positively charged PDMAEMA disrupts the bacterial membrane and enters the interior through strong multivalent electrostatic interactions with the negatively charged membrane, leading to highly efficient and selective antibacterial effects (Figure 1) (Zhang et al., 2022).

#### 2.1.2 Ligand-receptor–based specific recognition

The interactions between immune cells, the complement system, and bacterial surface structures are crucial for pathogen recognition and clearance by the immune system (Rivera et al., 2016; Lu et al., 2017a; Askarian et al., 2018). *Porphyromonas gingivalis* (*P. gingivalis*), a Gram-negative anaerobic bacterium, produces a variety of virulent factors that facilitate its adhesion, colonization, and nutrient uptake (Mysak et al., 2014). *Porphyromonas gingivalis* binds to Toll-like receptors (TLRs) through lipopolysaccharide (LPS) on its surface, triggering an inflammatory signaling cascade and the release of proinflammatory cytokines, ultimately leading to an inflammatory response (Tsukamoto et al., 2018). The virulence factors of *P. gingivalis* trigger localized inflammation, prompting the host’s immune system to restore periodontal health by primarily producing antibodies and engaging in phagocytosis (Lunar Silva and Cascales, 2021; Blasco-Baque et al., 2017). However, *P. gingivalis* evades immune surveillance by activating mechanisms that impair immune cell function, particularly macrophages. This ability disrupts the homeostasis between the host and its microbiota, altering the composition of the subgingival biofilm, promoting inflammation, tissue damage, alveolar bone loss, and ultimately leading to periodontal disease.

**FIGURE 2:**
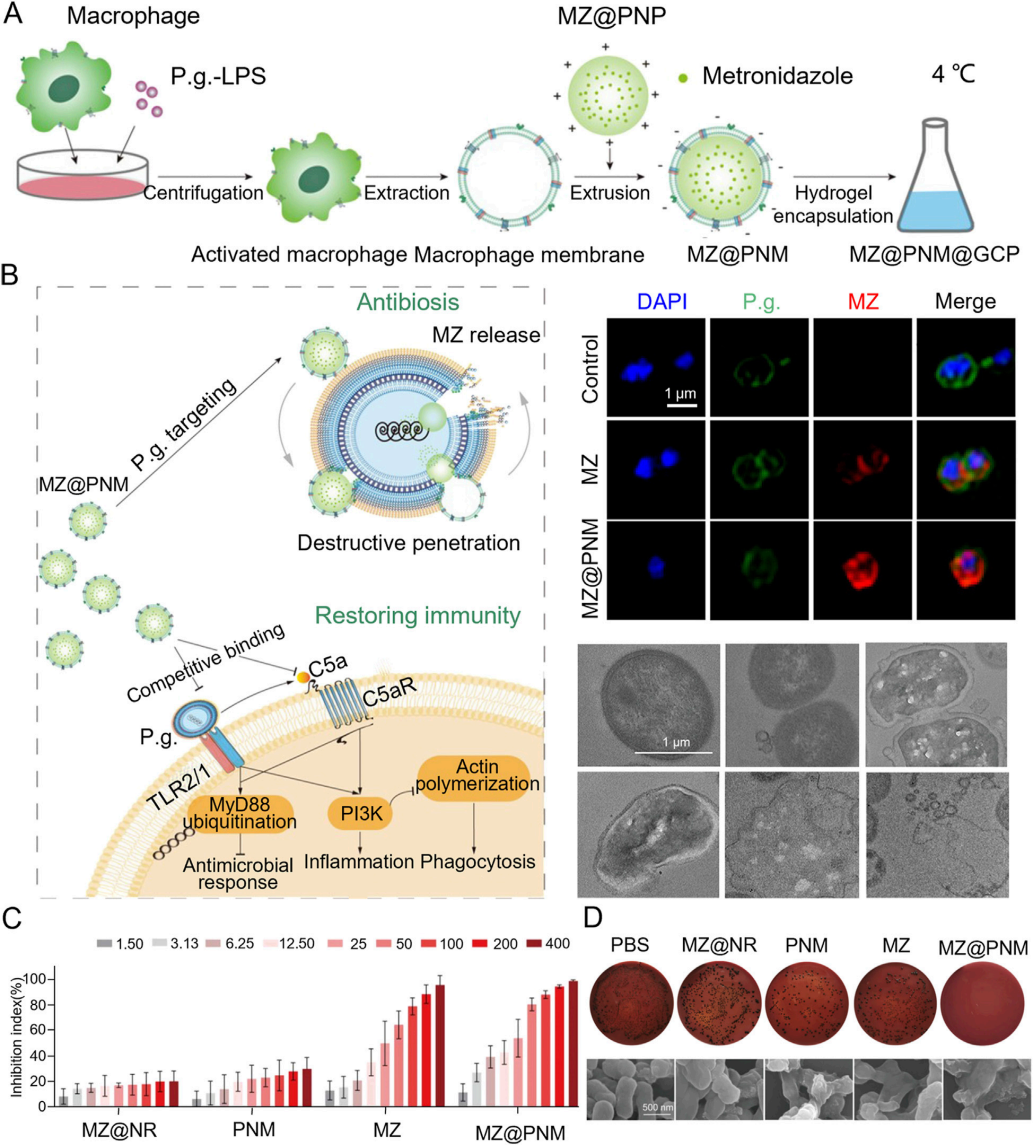
**(A)** Schematic diagram of the MZ@PNM@GCP hydrogel for periodontitis treatment. **(B)** Representative fluorescence images of *Porphyromonas gingivalis*. Incubated with MZ@PNM or MZ and TEM images of the destruction of *Porphyromonas gingivalis* by MZ@PNM **(C)** Proportion of *Porphyromonas gingivalis* inhibited after 24 h of incubation with MZ@NR, PNM, MZ and MZ@PNM, respectively. **(D)** Colony-forming assay of *Porphyromonas gingivalis* after treatment with PBS, MZ@NR, PNM, MZ and MZ@PNM, respectively (Yan et al., 2022). Copyright ^©^ 2022 American Chemical Society.

**FIGURE 3:**
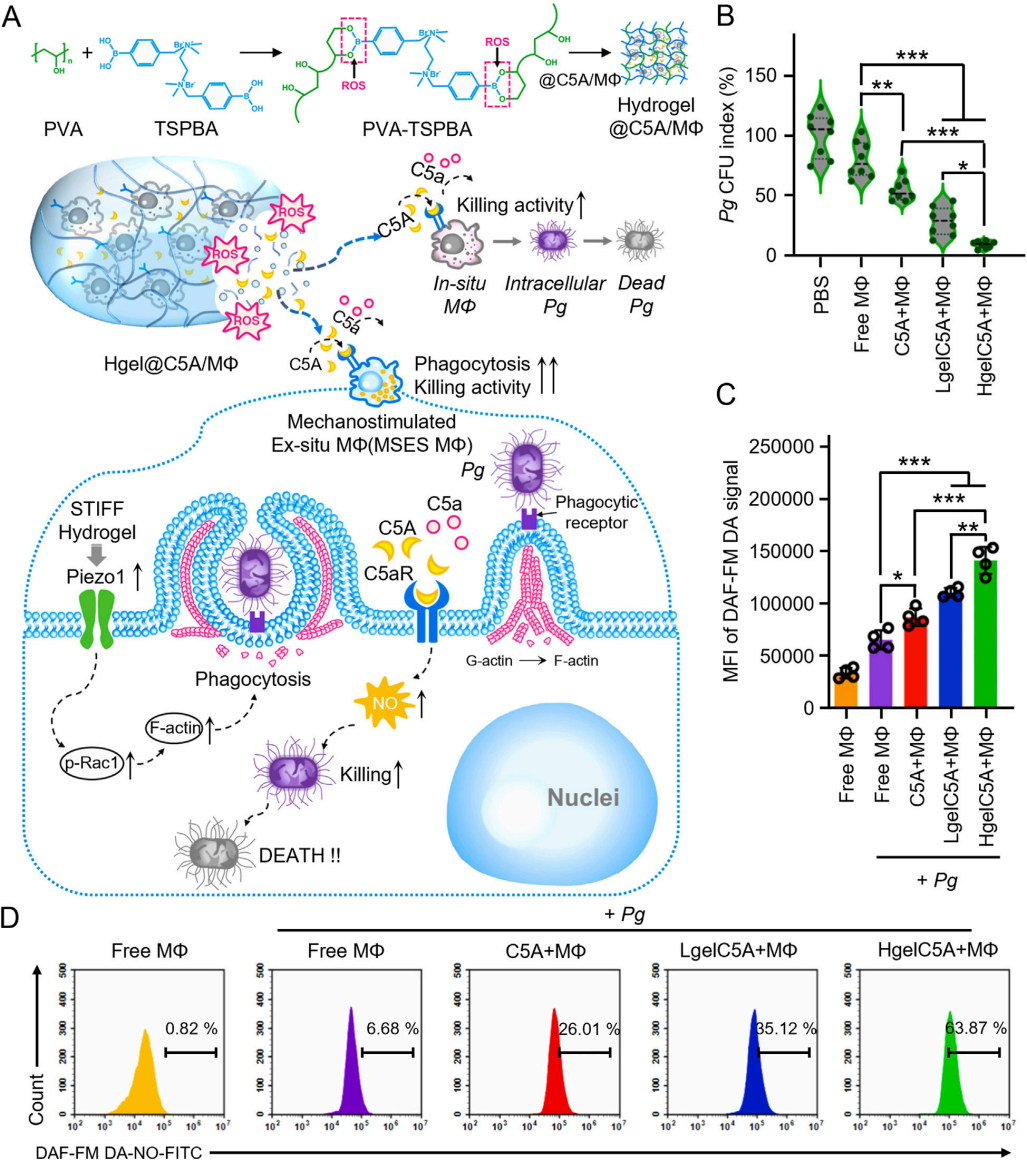
**(A)** Schematic illustration of mechanism how the high modulus ROS-sensitive hydrogel encapsulating macrophages and C5aR antagonists (Hgel@C5A/MФ) treats periodontitis. **(B)** Quantitative analyses showing the colony forming units (CFU) of residual live bacteria (n = 8, mean ± SD). **(C)** Quantitative analyses and flow cytometry **(D)** of NO production by RAW264.7 cells receiving different treatments (n = 4, mean ± SD) (Gan et al., 2023).

*Porphyromonas gingivalis* typically inhibits macrophage phagocytosis and bactericidal activity through TLR2/1 and complement C5a receptor (C5aR)-dependent signaling pathways. In response, researchers developed a microenvironmentally responsive nanogel (MZ@PNM@GCP) that mimics macrophage membranes, achieving a stealth effect. MZ@PNM@GCP specifically targets *P. gingivalis* through the TLR2/1 complex on the macrophage-mimicking membrane, disrupting the bacterial membrane with cationic nanoparticles and releasing metronidazole inside the bacterial cell. Encapsulating the nanoparticles in a responsive hydrogel allows controlled drug release in localized acidic and inflammatory environments, enhancing treatment efficacy. Additionally, the nanogel prevented *P. gingivalis* from binding to immune cells, restoring local immune function and targeting pathogenic bacteria. This approach has shown promising results in the prophylactic treatment of periodontitis (Figure 2) (Yan et al., 2022). Innate immune cells utilize TLR4 receptors to detect lipopolysaccharide (LPS) from bacteria, triggering an inflammatory response. Leveraging this TLR4 mechanism, researchers designed a novel nanoplatform (MSNCs) that mimics macrophage membrane properties and expresses TLR4, combining antimicrobial and immunomodulatory effects. By engineering immunocompetent cell membranes, MSNCs selectively localize to bacteria through TLR4 and act as molecular decoys, competitively binding to LPS in the microenvironment (Deng et al., 2023). Actin is a critical component of the cytoskeleton, involved not only in sensing and transmitting mechanical signals but also in macrophage phagocytosis of pathogens. To effectively eliminate *P. gingivalis* hiding within inactivated immune cells, researchers optimized hydrogel stiffness by adjusting the polyvinyl alcohol (PVA) cross-linking degree. This modification enabled targeted delivery of macrophages and C5a receptor antagonists to the gingival sulcus. At the molecular level, the stiffer hydrogel enhanced the expression of the mechanosensor Piezo1, which translates mechanical stimuli into biochemical responses, such as Rac1 activation and cytoskeletal reorganization, resulting in improved endocytosis and phagocytosis (Figure 3) (Gan et al., 2023).

The early colonization of the oral cavity by *Porphyromonas gingivalis* (*P. gingivalis*) is facilitated by interactions between its secondary hair antigens and oral streptococci, culminating in specific bacterial adhesion. *Streptococcus gordonii* typically serves as the initial colonizer, using its adhesion proteins to establish a foothold. These proteins provide a nutrient supply and a base for the secondary colonization of *P. gingivalis*, making *Streptococcus gordonii* an ideal target for therapeutic interventions. Researchers have engineered surface-modified PLGA nanoparticles (BNP) by incorporating a peptide (BAR) derived from *S. gordonii* surface proteins. This modification enhanced specific adhesion to *P. gingivalis* and prevents non-specific bioadhesion, leveraging the bacteria’s known interaction to initiate periodontal infections. This approach aimed to enable multivalent targeting and inhibit bacterial adhesion, as well as the formation of early biofilms (Mahmoud et al., 2019). Similarly, nanoparticles (ZnO_2_/Fe_3_O_4_@MV) were developed by encapsulating ZnO_2_ within Fe_3_O_4_ composite core-shell structures and coating them with *S. gordonii* membranes. This targeted membrane coating enhances nanoparticle internalization, where the combined action of hydrogen peroxide (H_2_O_2_) and hydroxyl radicals disrupts bacterial structures, ultimately leading to bacterial cell death and the removal of symbiotic biofilms (Cao et al., 2023).

The introduction of exogenous substances modulate bacterial subcellular organization by inducing intracellular aggregation through ligand-receptor interactions. This aggregation shows a strong affinity for various proteins, thereby disrupting bacterial contents. A novel method has been proposed for global bacterial disruption, based on DNA-induced intracellular aggregation. This method involved synthesizing dAPM-1, a di-arginine peptide mimetic with a specific spacer linkage, to trigger cellular cohesion through nuclear protein-DNA phase separation. This process disrupted subcellular tissues and induced membrane rupture, interfering with bacterial functions and inhibiting drug resistance (Yang et al., 2024).

### 2.2 Mechanisms of oxidative damage

Reactive oxygen species (ROS) exert antimicrobial effects primarily through oxidative damage, which undermines bacterial antioxidant defenses. ROS initiate lipid peroxidation by reacting with bacterial membrane lipids, increasing membrane permeability and compromising membrane integrity. This disruption leads to the leakage of bacterial contents and cell death (Elian et al., 2024). The H_2_O_2_ produced from ROS interactions with unsaturated fatty acids diffuses within the bacterial cell, interacts with proteins, and facilitates the penetration of metal ions or oxidized molecules, exacerbating lipid peroxidation and accelerating bacterial death (Liu et al., 2022a; Wang et al., 2020). Additionally, ROS oxidatively modify amino acid residues in proteins, altering their structure and function, which leads to protein inactivation. ROS also cause DNA strand breaks and base modifications, resulting in genetic mutations and abnormal gene expression, contributing to bacterial death (Pannunzio and Lieber, 2017; Liang et al., 2019; Srinivas et al., 2019).

Photodynamic therapy (PDT) is a promising bactericidal approach that utilizes photosensitizers to generate reactive oxygen species (ROS) for bacterial eradication (Zhang et al., 2020; Yu et al., 2023). ROS production occurs through two primary pathways: type I and type II (Liu et al., 2022b). The type I pathway generates superoxide anion radicals (O_2_•^−^) and hydroxyl radicals (OH•) through electron transfer, while the type II pathway produces singlet oxygen (^1^O_2_) through energy transfer between the photosensitizer and oxygen, with H_2_O_2_ as an intermediary (Chen et al., 2021). These highly reactive species interact with biological macromolecules, such as purine bases, specific amino acids, and mitochondrial membranes in DNA, resulting in oxidative damage that ultimately causes bacterial cell death (Di Mascio et al., 2019; Ran et al., 2022).

**FIGURE 4:**
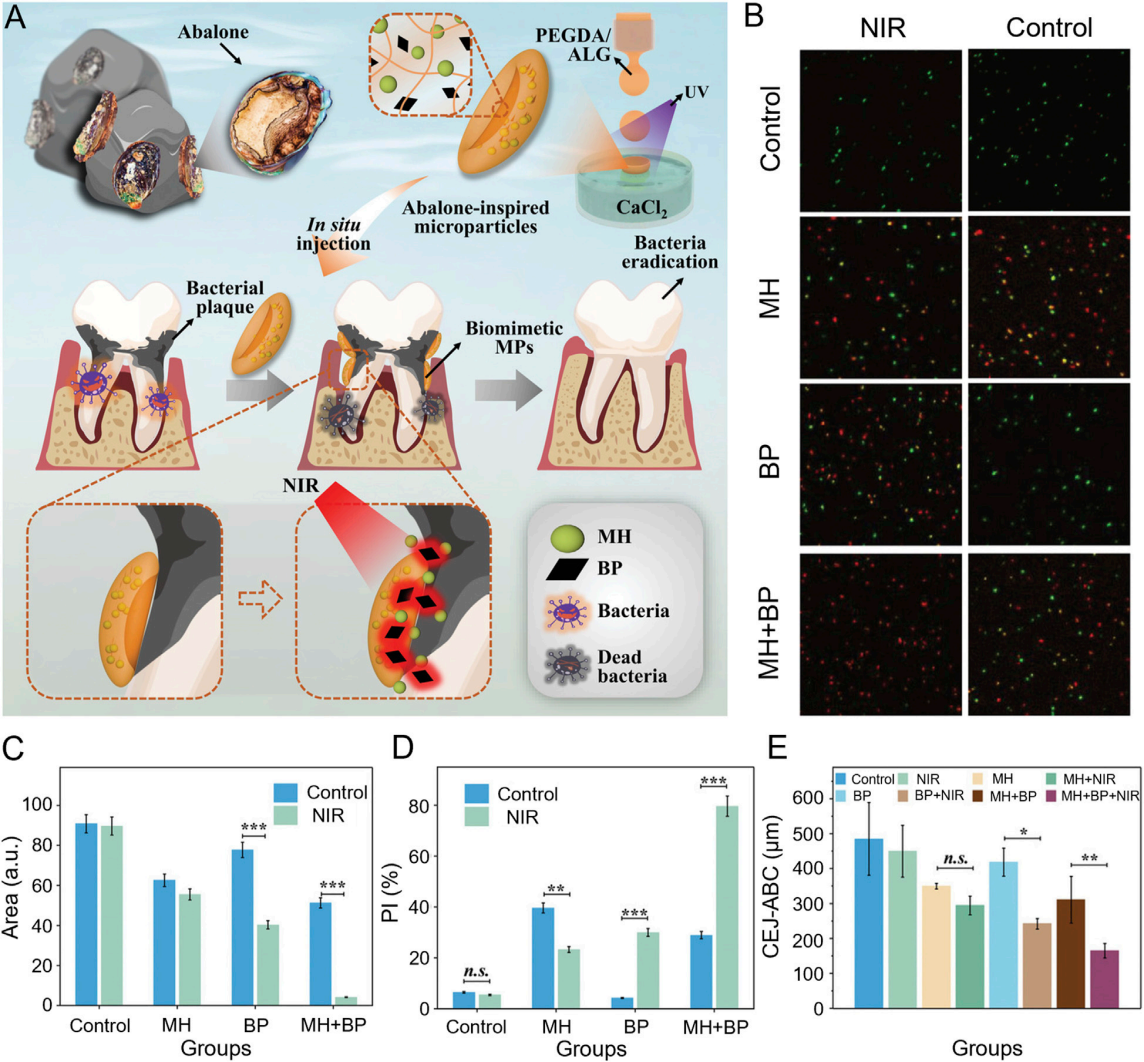
**(A)** Schematic illustration for the preparation and the anti-periodontitis mechanism of abalone-inspired microparticles. **(B)** Confocal images of the plaque biofilms stained with SYTO-9 and PI after various treatments. **(C)** The quantitative analysis of fluorescent images in **(B)** through Fiji. **(D)** The quantitative analysis of colony formation. (n = 3. ***p < 0.001). **(E)** The quantitative analysis of CEJ-ABC. (n = 3. **p < 0.01, *p < 0.05) (Song et al., 2022).

Advancing photosensitizer development is crucial for enhancing antimicrobial effectiveness. Under laser irradiation, the high density of positive charges on the brush layer of star-shaped polycationic brushes (sPDMA) significantly enhanced the binding of the photosensitizer indocyanine green (ICG) to bacterial membranes and its ability to penetrate biofilms. sPDMA@ICG NPs effectively killed *Porphyromonas gingivalis*, inhibited alveolar bone resorption, and reduced the inflammatory response in both *in vivo* and *in vitro* models (Shi et al., 2021b). Inspired by the abalone’s unique suction cup structure, Song et al. employed microfluidic electrospray technology to create a novel, adhesive, light-responsive particle (MP) delivery system for periodontitis treatment. The concave structure of the disc-shaped MPs confers enhanced adhesion and stability in the presence of saliva, while controlled release of minocycline hydrochloride and black phosphorus under near-infrared irradiation provides potent antimicrobial effects against *P. gingivalis* (Figure 4) (Song et al., 2022). Type I photosensitizers are not constrained by the periodontium’s local anoxic environment, reducing the dependence on oxygen during treatment. In the presence of light, the purine-based C^N ligand in the Ir(III) complex undergoes an n-π* transition, transferring energy to the Ir core and facilitating strong spin-orbit coupling. This mechanism promoted excited state transitions, enhancing the ROS-generating capability of the complex to kill anaerobic bacteria (Ding et al., 2024). An anaerobic cationic polymer (HQRB-SS-Dex) containing the photosensitizer rose Bengal (RB) and dextran, functions through both type I and type II mechanisms. The positively charged quaternary ammonium salts and dextran improved the photosensitizer’s surface adhesion and permeability to bacterial biofilms, particularly against anaerobic periodontal pathogens. The introduction of disulfide bonds significantly improved the biosafety of this complex, making it a promising candidate for clinical treatment of periodontal infections (Qian et al., 2023).

Photothermal therapy (PTT) is a hyperthermic treatment that utilizes near-infrared (NIR) light absorbers to generate heat, effectively killing bacteria through laser-induced temperature elevation (Li et al., 2020). The antimicrobial mechanisms of PTT include: (1) the conversion of absorbed light energy into thermal energy by nanomaterials, which rapidly raise the temperature and create a localized high-temperature environment; and (2) the high temperature increasing bacterial cell membrane permeability and directly damaging the bacterial cell wall, facilitating the penetration of antimicrobial drugs or photosensitizers, and leading to cellular content leakage. By designing nanomaterials with specific binding properties to the bacterial surface, PTT enhances therapeutic efficacy while minimizing host cell damage. Moreover, PTT may synergize with antimicrobial agents by promoting immune cell infiltration through thermal effects, thereby boosting the host immune response. Due to its physical mode of action, PTT presents a reduced risk of bacterial resistance, positioning it as a promising antimicrobial strategy, particularly against drug-resistant strains.

Zhang et al. developed an innovative nano-antibiotic delivery system that integrates gold nanocages (GNCs) with two temperature-sensitive components: phase change materials (PCMs) and the heat-sensitive polymer poly (N-isopropylacrylamide-co-bis-diethylaminomethyl methacrylate) (PND). This hybrid system formed a novel, light-triggered antibiotic platform, TC-PCM@GNC-PND. The heat-responsive properties of GNCs enable *in situ* formation of injectable hydrogels, enhancing local retention of tetracyclines (TCs) after their release at the infection site. Furthermore, the incorporation of positively charged tertiary amine groups in the PND facilitates targeted delivery to negatively charged bacterial surfaces. The efficient photothermal conversion of GNCs induced bacterial damage through heat-mediated membrane disruption and protein denaturation under near-infrared (NIR) irradiation, demonstrating potent antimicrobial activity. This process also triggered phase transitions in the PCM and contraction of the PND, providing precise control over the on-demand release of TCs. *In vitro* and *in vivo* studies confirmed the platform’s high bactericidal efficacy and low toxicity, offering valuable insights for the design of new antimicrobial materials (Zhang et al., 2024).

Sonodynamic therapy (SDT) employs ultrasound (US) to activate reactive oxygen species (ROS) generated by acoustic sensitizers, producing toxic effects on a broad spectrum of bacteria. This non-invasive technique offers deep tissue penetration, excellent time precision, and avoids the development of bacterial resistance. A novel acoustic sensitizer was developed by incorporating titanium dioxide (TiO_2_) onto dendritic mesoporous silica nanoparticles (DLMSNs), reinforcing the structure with silver (DT-Ag) and modifying it with quaternary chitosan (DT-Ag-CS). Upon returning to the ground state, TiO_2_ transfers energy to oxygen and water, generating ROS that serve as antimicrobial agents. Additionally, the collapse of cavitation bubbles produced localized high temperatures, promoting water pyrolysis and generating more hydroxyl radicals (Xin et al., 2023). This mechanism enhanced the antimicrobial efficacy of the sensitizer. Furthermore, the novel acoustic sensitizer TPP-TeV, a combination of tetraphenylporphyrin and telluric violet alkaloids, produced a substantial number of cationic radicals and ROS through electron transfer under ultrasonic radiation. This effectively killed anaerobic *P. gingivalis*, improving the local periodontal microbial environment and offering a promising new approach for SDT in the treatment of periodontitis.

The bacterial antioxidant system maintains the balance of ROS levels. When ROS production surpasses the scavenging capacity, oxidative stress ensues, potentially damaging cell membranes and proteins (Redza-Dutordoir and Averill-Bates, 2016). In some cases, dynamic regulation of ROS is essential to preserve physiologically necessary ROS while eliminating cytotoxic ones. For example, on-demand regulation of ROS can be achieved by modulating the surface state of carbon dots, allowing for efficient and precise treatment of chronic inflammation and infection (Nie et al., 2024). As a traditional antimicrobial agent, copper’s contact-killing mechanism plays a critical role in its antimicrobial effect, disrupting bacterial cell membranes. Furthermore, exposure to high concentrations of copper surfaces results in copper ions penetrating the membrane, where they rapidly kill bacteria by impairing respiratory activity and DNA integrity—causing DNA fragmentation and inhibiting respiration (Portelinha et al., 2021; Mahmoudi et al., 2022; Xue et al., 2023). The novel TM/BHT/CuTA hydrogel system integrated antioxidant properties with the antimicrobial activity of tannin-ligated copper nanosheets. This system efficiently delivered therapeutics to inflamed periodontal areas through electrostatic adsorption and physical adhesion. The intelligent release mechanism endowed the material with multiple ROS-scavenging capabilities and robust antimicrobial properties, showing great potential for periodontal treatments (Xinyu et al., 2023). In addition to its direct antimicrobial effects, copper ions generated harmful hydroxyl radicals through a Fenton-like reaction. ROS production not only covalently damages biomolecules but also depletes bacterial antioxidants. To enhance efficacy, amino groups were introduced into mesoporous silica (MSN)-coated citrate-grafted copper sulfide (CuS) nanoparticles (CuS@MSN), imparting a positive charge. These nanoparticles then interacted electrostatically with sulfated chitosan (SCS) to form CuS@MSN-SCS nanoparticles. Cu^2+^-mediated activation of the ROS signaling pathway enabled efficient bacterial eradication. The initiation of oxidative stress within *Fusobacterium nucleatum*—including DNA damage, protein oxidation, and lipid peroxidation—induced bacterial apoptosis and biofilm inhibition (Chen et al., 2024).

Nitric oxide (NO) plays a pivotal antimicrobial role in organisms, primarily through the generation of nitroso compounds and the induction of oxidative stress. These reactions disrupt bacterial functions while protecting host cell membranes. Notably, low concentrations of NO, which are not toxic to bacteria, effectively prevent biofilm formation and depolymerize established biofilms via cell signaling mechanisms. Moreover, macromolecule-based NO release systems exhibit superior antimicrobial effects due to enhanced NO loading and stronger binding affinity to bacteria. Under aerobic conditions, NO release significantly boosted the anti-biofilm activity of hyperbranched polymers. This enhancement is evident not only in reduced biofilm metabolic activity but also in the effective killing of bacteria isolated from the biofilm. NO’s excellent aqueous solubility, coupled with its dose-dependent anti-biofilm properties, presents the potential for incorporation into oral rinses, gels, or ointments, opening new avenues for the treatment of oral diseases (Yang et al., 2020).

### 2.3 Synergistic antibacterial effect

Single antimicrobial mechanism has a certain inhibitory effect against bacteria, but for multiple mechanisms synergistic antimicrobial can overcome the shortcomings of single antimicrobial to achieve more efficient antimicrobial effect. The synergistic antimicrobial action of bacterial membrane disruption and oxidative stress mechanisms against different bacterial targets not only increases the antimicrobial spectrum, but also prolongs the duration of action and reduces the likelihood of bacterial target escape (Hu et al., 2022).

**FIGURE 5:**
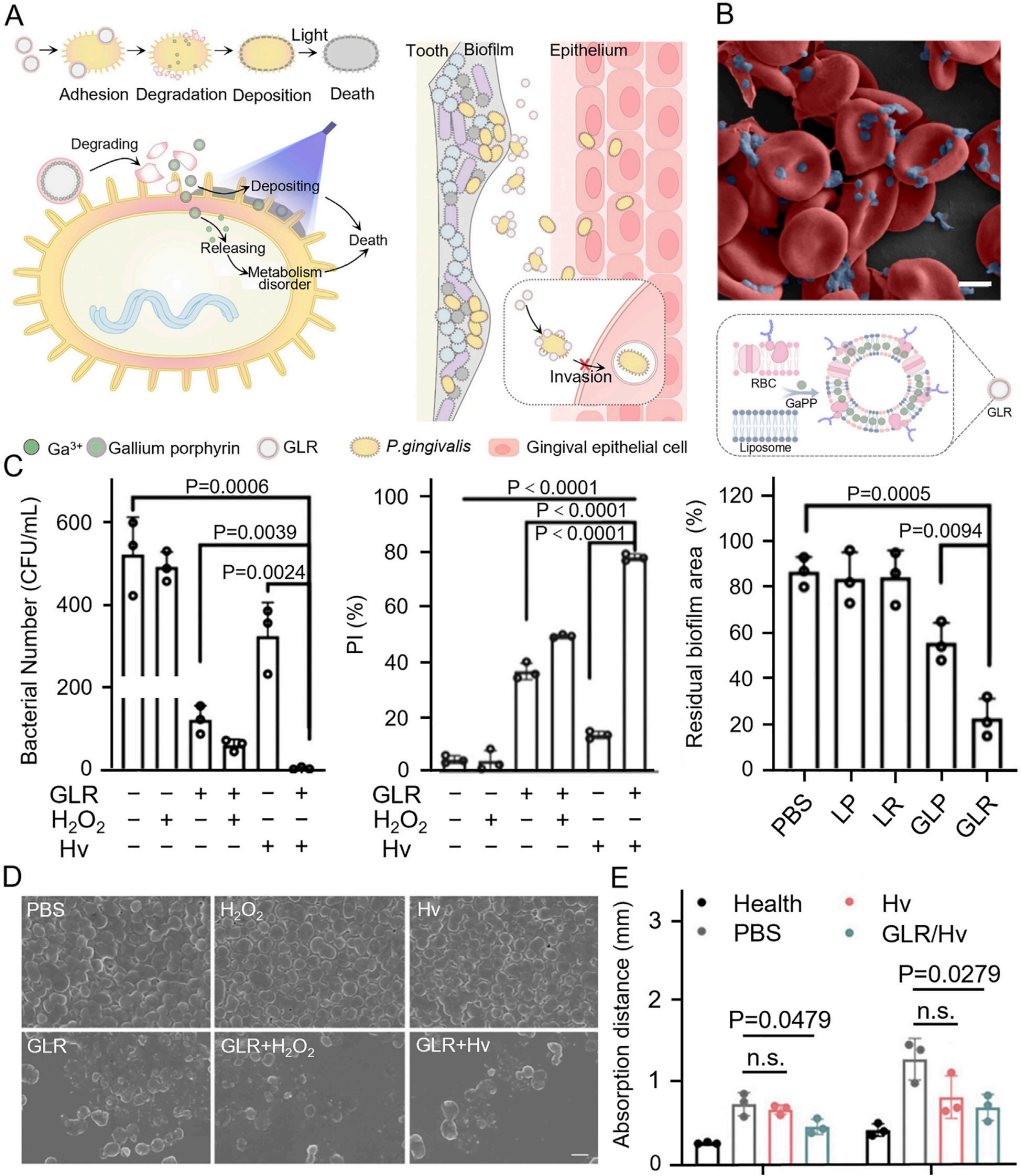
**(A)** GLR combined with *Porphyromonas gingivalis* and was cleaved, loading gallium porphyrin resulted in bacterial death through photodynamic therapy as well as disruption of bacterial metabolism, weakening bacterial invasion of epithelial cells. **(B)** SEM image of *Porphyromonas gingivalis* adhesion with RBCs and illustration of GLR. **(C)** Comparison of the antibacterial effect of GLR against *Porphyromonas gingivalis*. Under different treatments and relative quantification of the area of human subgingival biofilm residue. **(D)** SEM images of *Porphyromonas gingivalis* under different treatments. **(E)** Quantification of the distance of periodontal bone resorption (from the alveolar crest to the enamel-osteum junction) after periodontitis treatment in rats (Tang et al., 2024). Copyright ^©^ 2024 American Chemical Society.

*Porphyromonas gingivalis* acquires essential iron and heme from hemoglobin through hemagglutinin-mediated erythrocyte aggregation and protease hydrolysis, both crucial for its growth and virulence. Some heme is degraded by *P. gingivalis* proteases and transported to the bacteria via HmuR or HmuY receptors, while the excess accumulates on the bacterial surface. This excess heme contributes to the bacterium’s oxidative stress resistance through its peroxidase activity (Cao et al., 2024; Olczak et al., 2005; Aleksijević et al., 2022). To exploit this mechanism of adhesion to erythrocytes and heme utilization, Tang et al. engineered GLR (an erythrocyte membrane liposome loaded with gallium porphyrin) nanovesicles mimicking erythrocytes, loaded with gallium porphyrin to target the bacteria. Gallium ions from GLR disrupted bacterial metabolism, while surface-deposited porphyrins generate ROS through photodynamic therapy. This results in a synergistic antimicrobial effect both inside and outside the bacterium through distinct mechanisms (Figure 5A). Additionally, the increased sensitivity to oxygen enhanced antimicrobial effects. SEM revealed that *P. gingivalis* exhibited stronger adhesion to erythrocytes (Figure 5B). Treatments of *P. gingivalis* with this material maintained robusted antimicrobial efficacy even at low hydrogen peroxide concentrations, significantly reducing bacterial resistance to oxidative stress. The combination of GLR and photoirradiation (Hv) demonstrated a synergistic antimicrobial effect, effectively eliminating both suspended bacteria and biofilms (Figure 5C). SEM images confirmed the disruption of the bacterial membrane structure by GLR (Figure 5D). In animal studies, the treated rats exhibited a significant reduction in bone resorption, confirming the *in vivo* antimicrobial efficacy and the practical application potential of this approach (Figure 5E) (Tang et al., 2024).

The combination of bacterial membrane disruption, oxidative stress, and antimicrobial mechanisms exhibited enhanced antibacterial and anti-biofilm effects. Surfactin, an amphiphilic biosurfactant derived from *Bacillus subtilis*, contains a cyclic heptapeptide and β-hydroxy fatty acid structure. Nanoparticles loaded with various concentrations of surfactin inhibited periodontal pathogens and oral bacteria by modifying bacterial membrane hydrophobicity and inducing oxidative stress (Johnson et al., 2020; Johnson et al., 2021). Amphiphilic cationic polymers with guanidine groups readily interact with proteins, nucleic acids, and phospholipids, facilitating deep penetration and efficient bacterial adhesion. A charge reversal strategy temporarily neutralized the positive charge of these polymers to minimize toxicity, while the polymer’s acid-responsive release of the guanidine group reactivates its antimicrobial function when accumulated in biofilms. Upon near-infrared laser irradiation, photothermal agents (CS) encapsulated in the polymer generate heat, effectively eliminating bacterial biofilms.

Similarly, combining various pathways to generate oxidative stress has a more than twofold synergistic effect. Novel Bi_2_ S_3_/Cu-TCPP Z-type nanocomposites exhibited superior light absorption and efficient electron-hole separation. Theoretical calculations showed that the heterogeneous structure of Bi_2_ S_3_/Cu-TCPP facilitates the adsorption of oxygen molecules and hydroxyl radicals at its interface, enhancing ROS generation. PTT with Bi_2_S_3_ nanoparticles promoted Cu^2+^ ion release, augmenting the chemodynamic therapy (CDT) effect. Moreover, released Cu^2+^ ions deplete intracellular glutathione, weakening the bacterial antioxidant defense. The combination of PDT/PTT/CDT synergistically enhanced antimicrobial activity against periodontal pathogens and promotes biofilm eradication (Kong et al., 2023). By leveraging the catalytic effects of nano-enzymes and the photosensitization of self-oxygenating PDT materials, Sun et al. developed hybrid nanoplatforms that selectively target anaerobic bacteria, demonstrating exceptional antimicrobial activity and therapeutic selectivity. Additionally, the MnO_2_ nanolayer was modified to provide a continuous oxygen supply, addressing the challenges posed by the anaerobic microenvironment. This modification alleviated the hypoxic conditions in periodontal pockets and boosts the production of reactive oxygen species (ROS), significantly enhancing the therapeutic efficacy of PDT (Sun et al., 2021).

### 2.4 Other strategies

Embelin (Emb), a plant-derived compound, was successfully released in a controlled manner using a carboxymethyl chitosan oxidized dextran (CMCS-OD) hydrogel as a drug carrier. This was achieved through a dual dynamic network formed by ligand and Schiff base bonds. Molecular docking studies revealed that Emb interacts with efflux pump proteins, inhibiting their function by hydrogen bonding to their active sites. This interaction reduced the efflux of antimicrobial drugs, thereby influencing bacterial DNA gyrase/topoisomerases. Furthermore, Emb was shown to disrupt bacterial quorum sensing (QS), inhibiting the synthesis of virulence factors and biofilm formation. This reduced bacterial pathogenicity and enhanced the efficacy of antimicrobial drugs, potentially advancing a targeted antimicrobial strategy for the treatment of periodontitis (Cai et al., 2024a).

Bacteria within biofilms are organized in a structured extracellular matrix and interact through quorum sensing (QS) mechanisms. Intercellular communication regulates bacterial behavior and plays a pivotal role in biofilm formation (Bodelón et al., 2016; Su et al., 2023). Co-polymerization among pathogenic bacteria disturb the oral microbiota’s physiological balance, and the proliferation of biofilms is a significant contributor to microbiota dysbiosis. To restore ecological balance, cationic dextran was utilized to induce disruptions in the extracellular polymeric substances (EPS) matrix, promoting phase separation within 2 h and disrupting the matrix’s structural integrity (Li et al., 2023). Furazone C-30 acts as a disruptor of bacterial communication. When combined with Ca2+-coated PLGA particles and PBMP polymers, a novel PLGA/PBMP particle was developed. The sustained release of furazone C-30 from this particle effectively prevented biofilm formation, offering a promising strategy for preventing bacterial infections in periodontitis (Kang et al., 2019).

Probiotics offer a promising approach to address biofilm ecological dysregulation, with their antimicrobial action attributed to direct competition with pathogens for nutrients and adhesion surfaces. Beneficial strains isolated from the oral microbiota of healthy individuals were screened via genome sequencing for genes linked to antimicrobial and immunomodulatory activities, virulence factors, and antibiotic resistance transfer. The selected probiotics target specific periodontal pathogens without exhibiting cytotoxicity (Grilc et al., 2023). The application of oxygen to periodontal tissues exerts a toxic effect on anaerobic pathogens, significantly reducing bacterial colonization in both floating cultures and biofilms. Importantly, this process does not induce side effects or resistance. Oxygen also plays a critical role in energy production and cellular metabolism. Therefore, moderate oxygenation not only inhibits the growth of anaerobic bacteria but also stimulates angiogenesis, cell proliferation, collagen synthesis, and ultimately, periodontal regeneration. Ming et al. developed a biocompatible, oxygen-releasing thermosensitive hydrogel encapsulating small extracellular vesicles (sEVs) and calcium peroxide nanoparticles secreted by bone marrow mesenchymal stem cells (BMMSCs). This system enabled controlled release of sEVs and oxygen, effectively inhibiting the growth of anaerobic periodontal bacteria, alleviating anaerobic infections in periodontal pockets, and promoting the regeneration of periodontal defects (Ming et al., 2024).

## 3 Conclusions and future perspectives

This review examined polymer-based antibacterial strategies for treating periodontitis. By leveraging various antibacterial mechanisms—either individually or in combination—these strategies reduce the risk of drug resistance, providing insights for the development of future innovative therapies. While polymer-based antimicrobial therapeutics show significant potential in laboratory settings, their clinical efficacy remains to be fully validated. This step is crucial for optimizing and advancing novel therapeutic strategies.

Currently, most antimicrobial treatments for periodontitis focus on eradicating all bacteria within periodontal tissues. However, certain beneficial bacteria, such as lactobacilli and specific streptococci, can counteract or inhibit periodontal disease promoters (Wang et al., 2022). Therefore, developing targeted nanosystems or biomimetic strategies with selective antimicrobial effects is critical. These strategies must protect beneficial flora and normal tissue cells from harm. Given the complex interactions between microbial communities and the immune system, future antimicrobial therapies using nano-delivery systems should aim to restore the oral microbiota’s homeostasis rather than eliminate all microorganisms. Similarly, while current treatments focus on generating ROS to induce oxidative damage, excessive ROS accumulation contributes to periodontal tissue damage. Moderate ROS levels, however, activate c-Jun N-terminal kinase, which in turn activates the transcription factor AP-1 and anti-apoptotic genes, aiding cell survival (Mittal et al., 2014; Tan and Suda, 2018). Thus, maintaining low ROS levels in periodontal tissues is vital for promoting tissue regeneration and optimizing antimicrobial efficacy.

Innovative approaches for periodontitis treatment are advancing rapidly, although most remain in preclinical stages. *In vitro* and *ex vivo* models do not fully replicate the complexity of human periodontitis. Combining polymers with drug delivery systems can extend drug residence time in the periodontal pocket and increase local drug concentrations, offering a promising route for clinical application. As effective adjunctive periodontitis therapies, treatments such as photodynamic photothermal are expected to improve periodontal health indicators and reduce the risk of drug resistance when combined with routine periodontal scaling in the clinic, thus creating a more comfortable and convenient periodontitis diagnosis and treatment process (Joshi et al., 2020). Currently, differences in experimental design such as different photosensitizers and laser wavelengths can lead to differences in the clinical indicators of the samples, and there is a need to further standardize the parameters and treatment specifications in order to achieve controlled and visualized efficient diagnosis and treatment (Chiang et al., 2020; Sukumar et al., 2020). Moreover, periodontitis is often associated with a range of comorbidities, including diabetes and hypertension (Bosi et al., 2013; Cai et al., 2024b). Therefore, future antimicrobial therapeutic strategies must consider the treatment of these comorbidities to ensure that new therapies are effective for patients with conditions associated with periodontitis.
